# Arteriovenous fistula as the vascular access contributes to better survival of hemodialysis patients with COVID-19 infection

**DOI:** 10.1177/11297298211021253

**Published:** 2021-06-03

**Authors:** Ahmet Murt, Serap Yadigar, Serkan Feyyaz Yalin, Mevlut Tamer Dincer, Ergun Parmaksiz, Mehmet Riza Altiparmak

**Affiliations:** 1 Department of Nephrology, Cerrahpasa Medical Faculty, Istanbul University – Cerrahpasa, Istanbul, Turkey; 2 Department of Nephrology, Kartal Dr. Lutfi Kırdar City Hospital, Istanbul, Turkey

**Keywords:** COVID-19, hemodialysis, AVF, catheter, dialysis access

## Abstract

**Background:**

While COVID-19 in chronic hemodialysis patients has high mortality and the pandemic will not end in the near future, effective follow up strategies should be implemented for these patients. Surgeries have been triaged according to their level of urgencies and arteriovenous fistula (AVF) operations were among elective surgeries. This study aimed to analyze the effect of vascular access on the outcomes of hemodialysis patients who had COVID-19.

**Methods:**

One hundred four hemodialysis patients who had COVID-19 were retrospectively analyzed. Seventy-two of them had AVF as the vascular access while 32 of them had tunneled catheters. Inflammatory markers and outcomes of patients with AVFs and catheters were compared. A logistic regression analysis was performed in order to define factors that contribute to better outcomes in hemodialysis patients.

**Results:**

COVID-19 had high mortality rate in hemodialysis patients (36.5%). Patients with catheters have higher peak ferritin levels (*p* = 0.02) and longer hospital stay (*p* = 0.00). Having AVF as the vascular access (OR = 3.36; 95% CI: 1.05–10.72; *p* = 0.041) and using medium cut-off dialyzers (OR = 7.99; 95% CI: 1.53–41.65; *p* = 0.014) were related to higher survival of the patients. COVID severity was inversely proportional to the survival (*p* = 0.000)

**Conclusions:**

AVFs contribute to higher survival of hemodialysis patients with COVID-19. Even in the pandemic era, end stage renal disease patients should be given the opportunity to have their vascular access properly created.

## Introduction

While the novel coronavirus disease (COVID-19) is estimated to have 1%–3% of case fatality,^1^ patients who have weakened immune system may have higher risks for worse outcomes. Immune dysfunction is also a component of chronic kidney disease (CKD) with decreased dendritic cells and defective B and T cell functions.^2,3^ Additionally pro-inflammatory cytokines and inflammatory monocytes are generally increased. Thus, CKD patients are at increased risk for infectious diseases and once infected, COVID-19 may have more severe course in these patients.

Hemodialysis is the most prevalent renal replacement modality^4,5^ and Kidney Disease Outcomes Quality Initiative (KDOQI) clinical practice guidelines recommend arteriovenous access instead of central venous catheters (CVC) for hemodialysis.^6^ However, in their case triage guideline for COVID-19 pandemic, American College of Surgeons declared that AV fistula or graft operations were in the group of elective operations and postponing should be considered.^7^ A similar approach was also implemented in our country.^8^

This study aimed to study COVID-19 disease course in maintenance hemodialysis patients and to analyze if vascular access had an impact on the outcomes.

## Materials and methods

### Setting

Patients were enrolled from two tertiary care hospitals (Cerrahpasa Medical Faculty and Kartal Dr. Lutfi Kirdar City Hospital) located in Istanbul, both of which served as pandemic hospitals. Istanbul has been the epicenter of the pandemic in Turkey. From 11th March 2020 to 1st February 2021, 2434 patients from Cerrahpasa and 8146 patients from Kartal were followed up in the inpatient COVID clinics.

### Patients and diagnosis

Maintenance hemodialysis patients who were hospitalized with COVID-19 were studied retrospectively. COVID-19 was diagnosed by real-time polymerase chain reaction (RT-PCR) for SARS-CoV-2 from combined oral and nasopharyngeal swabs or symptoms, laboratory tests, and chest computed tomography (CT) findings point out to COVID-19 diagnosis. Patients who were younger than 18 years old and those who use temporary hemodialysis catheters were excluded. A total of 104 patients were enrolled in the study.

### Descriptions

Patients who use a surgically created arteriovenous fistula (AVF) as the vascular access constituted the fistula group. Patients who use tunneled central venous catheters for hemodialysis formed the catheter group. All of the patients in the catheter group were using tunneled catheters for long term. There was not any patient who had arterio-venous graft.

COVID-19 disease activity during hospital stay was graded according to a scale that included asymptomatic infection (0 point), mild disease with upper respiratory tract or gastro-intestinal findings (1 point), COVID-19 pneumonia (2 points), COVID-19 pneumonia with hypoxemia (3 points), or severe COVID-19 with multi-organ involvement (4 points).

Complete blood count and inflammatory markers (CRP and ferritin) were followed up on daily basis. On admission laboratory values as well as peak levels of the inflammatory markers were noted. Duration of hospital stay and intensive care unit admission (ICU) and mortality rates were computed.

### Lung involvement rates and COVID severity

Ninety-five patients had pulmonary involvement in chest CT scans. COVID severity frequencies were as follows: 1 patient had asymptomatic infection (1%), 9 patients had mild disease (8.7%), 43 patients had pneumonia without hypoxemia (41.3%), 38 patients had pneumonia with hypoxemia (36.5%), 13 patients had severe disease with multi-organ involvement (12.5%).

### Hemodialysis sessions

Three times weekly routine hemodialysis (HD) sessions were resumed for hemodialysis patients in isolated HD units. Each HD treatment was applied for 4 h a day. Thirty-seven patients under-went HD using medium cut-off dialysis membranes (Theranova 400, Baxter™, Deerfield, IL, USA) and 67 patients had HD with low flux membranes (Elisio-21 M, Nipro™, Osaka, Japan). The dialysate contained the following ion concentrations: Na 140 mmol/L, K 2–3 mmol/L, Ca 1.25–1.50 mmol/L, Mg 0.5 mmol/L, and HCO_3_ 32 mmol/L (Renasol BA-310, Fresenius Medical Care, Turkey). Blood flow rates ranged between 300 and 400 mL/min. The dialysate flow rate was constant at 500 mL/min for each patient. Ultrafiltration was programmed according to the patient’s volume status. Heparin or low-molecular-weight heparins were used as anticoagulants.

### Treatment modalities

According to the guidelines released by Turkish Ministry of Health, different treatment modalities have been used including hydroxychloroquine, favipiravir, lopinavir/ritonavir, and/or anti-cytokine drugs such as steroids, anakinra, or tocilizumab.^9^ When needed, antibiotic coverage was also added to the treatment. Nasal oxygen, high-flow oxygen, or non-invasive mechanic ventilation support were provided depending on the oxygen saturations. Patients who have sustained hypoxemia despite oxygen therapies or multi-organ failure were admitted to intensive care unit. We prescribed prophylactic anticoagulants (enoxaparin 4000 IU subcutaneously) because of high rate of thromboembolic complications among hospitalized patients with COVID-19.

### Ethics approval

Both the institutional medical ethics committee in Cerrahpasa Medical Faculty (Nr. 21.04.2020/A-10) and the national COVID-19 scientific supervision committee (Nr. 2020-05-08T17_04_43) approved the study.

### Statistical analysis

Continued parametric data was presented as mean ± standard deviation and *t*-test was used for comparisons between groups. Categorical variables were expressed with percentages and were compared by chi-square or fisher’s exact test. A two-tailed *p* value less than 0.05 was accepted as statistically significant. Taking in-hospital mortality as the outcome measure, a multivariate logistic regression analysis was applied in order to define factors that contribute to higher survival. SPSS Statistics software version 22.0 (Chicago, IL) was used to carry out statistical analysis.

## Results

A total of 104 chronic hemodialysis patients were included in the study. Patients were 61.7 ± 13.9 years old and 51% of them were males. Patients spent 55.8 ± 40.4 months in hemodialysis. Among etiologies of their end stage renal diseases (ESRD), 29 patients (27,8%) had diabetic nephropathy, 26 patients (25%) had hypertensive kidney disease, 12 patients (11.5%) had glomerulonephritis, 8 patients (7.6%) had rheumatic diseases, 4 patients (3.8%) had polycystic kidney disease, 4 patients (3.8%) had secondary amyloidosis, 4 (3.8%) patients had post-renal pathologies, and 3 patients had malignancy associated kidney diseases (2.8%). Etiology of ESRD could not be defined in the remaining 14 patients (13.4%). Average duration of hospital stay was 11.9 ± 11.7 days. COVID-19 had high intensive care unit (ICU) admission rates and mortality among our hemodialysis patients, being 33.7% and 36.5% respectively. Seventy-two patients had AVF as the vascular access and 32 of them had tunneled catheters.

When patients using AVFs or catheters as the vascular access were compared; patients with catheters had lower albumin and lower hemoglobin levels. Peak ferritin and CRP levels, as markers of inflammatory response were measured higher for catheterized patients and on later days. Peak ferritin level was 3307.1 ± 7699.4 ng/mL (Median: 1065 ng/mL, median day: 7) in the catheter group while it was 1193.6 ± 923.2 ng/mL (Median: 944 ng/mL, median day: 4.5) in the AVF group. Peak CRP level was 217.4 ± 79.0 mg/L (Median: 210 mg/mL, median day: 7) in the catheter group and it was 179.9 ± 108.1 mg/L (Median: 172 mg/mL, median day: 3) in AVF group. COVID severity was not different between the groups. Co-morbidities were comparable between the groups. Kt/*V*, as an indicator of dialysis adequacy was also similar for both groups. Average time spent in dialysis was higher for patients with AVFs. Patients with catheters had longer hospital stay and their mortality reached as high as 50% (Table 1). When classified according to COVID-19 severity grades, proportion of patients with AVFs was the least and mortality was the highest in the severe COVID-19 group (Table 2).

**Table 1.** Comparison of patients with AVFs or catheters as the vascular access.

	Fistula (*N* = 72)	Catheters (*N* = 32)	*p*
Age	63.1 ± 12.3	58.6 ± 16.7	0.128
Gender (M/F)	39/33	14/18	
Diabetes (*n*, (%))	30 (41)	7 (21)	0.07
Hypertension (*n*, (%))	56 (77)	19 (59)	0.06
Other co-morbidities			
CHF/IHD (*n*, (%))	47 (65)	22 (68)	0.82
CVD (*n*, (%))	5 (7)	5 (15)	0.27
Malignancies (*n*, (%))	4 (5)	3 (9)	0.67
Mean arterial pressure (mmHg)	94.9 ± 11.8	95.8 ± 13.2	0.72
Albumin (g/dL)	3.5 ± 0.3	3.3 ± 0.5	0.01
Hemoglobin (g/dL)	10.3 ± 1.5	9.6 ± 1.3	0.03
Lymphocytes (per μL)	1064 ± 517	978 ± 680	0.47
Ferritin (ng/mL)	843.0 ± 766.1	1586.2 ± 3669.6	0.11
Peak ferritin (ng/mL)	1193.6 ± 923.2	3307.1 ± 7699.4	0.02
CRP (mg/L)	80.9 ± 63.0	94.3 ± 68.5	0.33
Peak CRP (mg/L)	179.9 ± 108.1	217.4 ± 79.0	0.08
Kt/V	1.67 ± 0.54	1.60 ± 0.74	0.55
Time spent in dialysis (months)	65.2 ± 40.5	34.9 ± 31.7	0.00
COVID severity	2.42 ± 0.80	2.72 ± 0.95	0.09
Lung involvement (*n*, (%))	68 (94)	27 (84)	0.23
Hospital stay length (days)	8.6 ± 3.9	19.0 ± 18.3	0.00
ICU requirement (*n*, (%))	22 (30.5)	13 (40)	0.37
In hospital mortality (*n*, (%))	22 (30.5)	16 (50)	0.07

AVF: arterio-venous fistula; M: male; F: female; CHF: congestive heart failure; IHD: ischemic heart disease; CVD: cerebrovascular disease; ICU: intensive care unit.

**Table 2.** Proportions of AVF versus catheters and mortality for different COVID-19 severity grades.

COVID-19 severity	Total number (*n*, (%))	AVF (*n*, (%))	Catheters (*n*, (%))	Mortality (*n*, (%))
0—Asymptomatic	1 (1)	1 (100)	0	0
1—Mild disease	9 (8.7)	6 (66.6)	3 (33.3)	0
2—Pneumonia	43 (41.3)	32 (74.4)	11 (25.6)	12 (27.9)
3—Hypoxemia	38 (36.5)	28 (73.6)	10 (26.3)	13 (34.2)
4—Multi-organ involvement	13 (12.5)	5 (38.4)	8 (61.5)	13 (100)
Total number	104 (100)	72 (69.2)	32 (30.7)	38 (36.5)

AVF: arterio-venous fistula; catheter: long term tunneled catheters; mortality: in-hospital mortality.

Refer to text for COVID-19 severity grades.

In a multivariate logistic regression analysis model with variables that had the highest likelihood to predict the outcome, fistulas as the vascular access and medium cut-off membranes as the dialyzers were significantly related to survival. Additionally, COVID severity was conversely proportional to the survival. On the other hand, age of the patients, peak levels of inflammatory markers (CRP and ferritin), or presence of co-morbidities, including diabetes and hypertension were not related to in-hospital mortality in our cohort (Table 3).

**Table 3.** Multivariate model to analyze factors that contribute to the outcome.

	Odds	95% CI	*p*
Age	0.96	0.92–1.03	0.068
Fistula	3.36	1.05–10.72	0.041
Medium cut-off membrane	7.99	1.53–41.65	0.014
COVID-19 severity	0.24	0.11–0.53	0.000
Peak ferritin	0.99	0.99–1.00	0.101
Peak CRP	1.00	0.99–1.00	0.33
Co-morbidities	1.60	0.10–25.09	0.73

CI: confidence interval.

## Discussion

COVID-19 has been previously shown to have high mortality (15%–25%) in CKD patients. Mortality was shown to increase with decreasing glomerular filtration rate (GFR) and was highest in dialysis population.^10^ This is most probably related to decreased immune responses in hemodialysis patients, which make them prone to infections. Overall mortality was also high in our cohort reaching as high as 36.5%. Such high mortality in hemodialysis patients necessitates proper implementation of infection control measures for this group.^11^

Nevertheless, there seems to be different factors, which affect the outcome of COVID-19 infection in hemodialysis patients. Our study showed that, medium-cut off dialyzers and vascular access of AVFs may contribute to better outcomes of COVID-19 patients. Positive effect of medium cut off dialysis membrane is to filter inflammatory mediators including IL-1β and IL-6. Medium cut-off membranes are quite promising and their benefits in COVID-19 patients have been reported before.^12,13^ COVID severity was the most important factor for mortality in our multivariate model whereas inflammatory markers and co-morbidities were not found to be significant. This might be because, high levels of inflammatory markers are related to COVID severity and hemodialysis patients already have a high burden of co-morbidities.

In the subgroup analysis of hemodialysis patients, patients with an AV fistula and those with a catheter were similar for age and co-morbidities. In spite of the similarity for COVID severity, peak CRP and ferritin levels were higher for patients in whom catheters were used as vascular access. Such higher levels in catheter group point out to higher inflammatory response in catheterized patients. Hemodialysis patients with higher inflammatory markers were previously shown to have higher mortality.^14^ Additionally these patients had lower albumin and hemoglobin levels, which might be other consequences of chronic inflammation. In hospital stay was significantly longer for patients with a catheter.

Because of the retrospective nature of the study, blood cultures were not available for all patients. However, patients with catheters might have experienced more secondary infections, which might have complicated the COVID-19 clinical course. Additionally, even non-infected catheters were previously shown to be associated with higher inflammation.^15^ So, infected or not, catheters might worsen COVID-19 which is a thrombogenic inflammatory disease. Such effects may prone hemodialysis patients with catheters to have longer hospital stay. One may speculate that, characteristics of the patients might have caused failed AVF attempts and these might be confounding factors for worse COVID-19 outcomes. However, age, history of diabetes, or vascular diseases, which might be related to AVF failures, were similar between the catheter and fistula groups. In a study from Wuhan, 29 of the COVID-19 patients died of vascular access complications and 70% of the complicated patients were using catheters as the vascular access for hemodialysis.^16^ Such findings underline the importance of AV access as a relevant preparation to cope with uncontrolled inflammatory and thrombogenic complications if a hemodialysis patient becomes infected with SARS-CoV-2. This is also in line with the KDOQI guideline, which states that an AV access should be preferred to a CVC in incident HD patients. According to our results, such an approach is also appropriate during COVID-19 pandemic and patients should be given the opportunity to have an AV access instead of a CVC. Khalid et al.^17^ shared their experiences for AVF surgeries and reported safe practices without major problems. Hence, every effort should be given in order not to delay AVF operations during COVID-19 pandemic and surgery organizations should re-consider their policies which recommend postponement for AVF operations. Deferral of AVF operations might have socio-economic, medical, and ethical consequences. Firstly, this will result in more frequent use of catheters for hemodialysis. Catheter related complications that will need longer hospitalizations might cause a higher economic burden. Additionally, vascular access would have an impact on survival of certain patient groups. The pandemic does not seem to end in the near future and ESRD patients should not miss proper vascular access opportunities. Balancing the use of resources with patient-centerd decisions sounds more reasonable throughout the pandemic.^18^ Timely planned hemodialysis with appropriate preparations can be an important measure to mitigate COVID-19 related mortality in patients with low GFR.

Small sample size may be a limitation of this study and larger studies may be needed to strengthen the results reached here.

## Conclusions

COVID-19 has high mortality in hemodialysis patients. As recommended for otherwise normal incident hemodialysis patients, AV fistula as the vascular access is better than venous catheters also for COVID-19 patients. We recommend that, hemodialysis planning should not be delayed for patients who have low GFR during COVID-19 pandemic and that relevant AV access (fistula) preparation has utmost importance for decreased mortality.
